# Mass Diffusion of Modern Digital Technologies as the Main Driver of Change in Sports-Spectating Audiences

**DOI:** 10.3389/fpsyg.2022.805043

**Published:** 2022-04-25

**Authors:** Ekaterina Glebova, Michel Desbordes, Gabor Geczi

**Affiliations:** ^1^CIAMS, Université Paris-Saclay, Orsay, France; ^2^CIAMS, Université d’Orléans, Orléans, France; ^3^Emlyon Business School, Écully, France; ^4^Department of Sport Management, Budapest University of Physical Education, Budapest, Hungary

**Keywords:** digital technology, sports spectacle, sports audience, sports consumption, sports transformation, sports digitalization, technological impact

## Abstract

The rapid uptake of digital technologies is constantly transforming the modern culture of sports spectating; however, relatively little is known about the impact of digitalization on the changing face of global sports-consuming audiences, particularly from a qualitative perspective. In this article, the relationship between modern mass digital technologies (i.e., mobile applications and big data) and audiences of sports spectators is described and explained by taking a customer-centric approach to grounded theory using a literature review and in-depth qualitative semi-structured interviews (*N* = 12) with sports marketing, management, and technology professionals. The qualitative approach permits the gathering of in-depth insights into a problem, generates new concepts through data synthesis and analysis, and captures changing attitudes within the sports industry. Moreover, the qualitative approach to research is not bound by the limitations of quantitative methods and focuses on the primary questions of “how” and “why” digital technology’s mass deployment and diffusion have transformed content consumption in the sports industry. The analysis first systematizes and codes the collected data. Second, all the materials are iteratively read and the key points are outlined. Using the iterative analysis, the theoretical and empirical insights and findings are synthesized in order to achieve the results. Finally, dimensions of the transformation of sports spectators’ consumption are identified and explained. The research implications highlight “how” and “why” modern digital technologies have changed the sports-consuming audience by making it more inclusive in terms of age, gender, demographics, social and health status. These findings are useful for sport managers to better understand their audiences and processes in an ever-changing global society.

## Introduction and Theoretical Background

The consumption of sports (Shank, 2005) is accompanied, invaded, and encompassed by various digital technologies that are gradually affecting the sports industry by touching all of its stakeholders, including the spectators who form the core of the sports ecosystem (Da Silva and Las Casas, 2017, 2018; Gruettner, 2019; Laukyte, 2020).

Modern technology has substantially transformed how sports are consumed and in some cases even created new platforms for participation and spectatorship (Kim et al., 2020). In this article, we aim to present technology as the main driver of change in sports-consuming audiences from a customer-centric perspective and understand and explain this process by outlining the key dimensions of this phenomenon.

### Sport Spectating as Experience and Digital Technologies

According to Rowe (2011), sport in general and sports spectatorship have emerged in the place-based experiential framework. Glebova and Desfontaine (2020) propose a construct of sports spectators’ customer experiences (SSCX), referring to a customer-centric approach and seeing a sports fan as a customer. The development of audiovisual media has divided the sports audience in two—the physically present audience that is engaged in a monetary exchange and the mediated audience that gains access without a significant charge. Tamir (2022) discloses the natural life cycle of a sport fan. Also, Rowe (2011) explains the “dual audience status” as the “viewer” and the “viewed,” meaning that an in-stadium audience can be considered part of the media sports audience because it has become accustomed to the benefits of mediatory watching that in some ways exceed those of physical attendance.

The sports media is of phenomenal appeal to global audiences (Lines, 2000). Communication technology allows out-of-town fans to watch any game and follow their favorite clubs. Accordingly, teams and leagues are expanding their marketing strategies globally—well beyond their geographical zones—and fans are able to affiliate strongly with distant teams (Lianopoulos et al., 2021). As a result of digital transformation on a global scale, new sports fan classifications are emerging (Giulianotti, 2002; Pu and James, 2017; Glebova et al., 2020). Notably, Nikolai (2020) proposes the term “dispersed fan” to embrace three types—distant fans, satellite fans, and displaced fans—in order to “describe the geographically distant populations of the global sports fan community,” pointing to the modern digitalized global sports fan community. In this regard, technology plays a significant role in shaping a fan’s identity (Allison and Knoester, 2021), categorization, salience, and distinctiveness (Shuv-Ami and Alon, 2020; Itonics, 2021). Using optimal distinctiveness theory as a framework, Brewer (1991) points out that individuals are motivated by the need for inclusion and the need for differentiation, which are a pair of fundamental and competing human needs (Shuv-Ami and Alon, 2020). However, Itonics (2021) now reports that the “multidimensional me” trend is blurring the traditional borders and dimensions between human and fan identity.

Furthermore, at the same time, Glebova et al. (2020) describe the changes in sports spectacle culture as “physical” and “virtual” relocations from the experiential perspective: relocations of SSCX are explained through the prism of technological development and the evolution of tools for the consumption of sports content. Internet connectivity, paired with digital technologies, lets spectators enjoy greater flexibility in terms of their viewing setting—which allows for a more flexible environment for discursiveness and socialization to take place—and advanced media, for example, allows for real-time analytics and instant replay. With the availability of sports content *via* multiple forms of telecasting and the Internet, sport fans can watch different kinds of televised sporting activities or follow their favorite teams and athletes from anywhere in the world (Glebova et al., 2020; Kim et al., 2020). The COVID-19 pandemic has even accelerated technologies mass deployment and gaming trends among global fans’ community (Glebova et al., 2022).

Laukyte (2020) notes that disruptive technologies affect the sports ecosystem involving fans and offer new possibilities. Wagner (2021) discusses the impact of online sports communities’ business on sport management. Poll (2019) explains that technology in general enables the greatest number of spectators to witness how sports can improve the lives and destinies of people, such as in the Paralympic movement.

### Gender Role

Two longitudinal studies (Meier et al., 2015) provide some evidence for the claim that the debate about feminization reflects changes in female fandom (although cultural shifts in values leading to an increase in numbers does not imply greater acceptance by male fans), adding to the knowledge on long-term trends in the feminization of sports. They also suggest that feminization reflects inauthentic consumerism and increases team identification as well. Furthermore, consistent age effects can be interpreted as indicating that the trends relate to changes in attitudes toward gender roles. Pope (2017) demonstrates that gender “performance” and “emphasized femininity” is a highly complex arena, providing a profound literature review on this topic. Furthermore, she examines quantitative approaches for “measuring” sports fans’ motivations and some of the contradictory findings in gender differences. Information and communication technologies (ICT) skills are found “to be gendered” (Koinig et al., 2020).

### Technology Acceptance and Age

Technology acceptance has become a key concept for the successful rollout of technical devices (Wilkowska and Ziefle, 2009). The technology acceptance model models how users come to accept and use a technology (Davis, 1989; Venkatesh et al., 2003), embracing perceived usefulness and perceived ease of use. The perception of technologies may vary depending on different variables. The effects of age and gender on technology acceptance ratings have also been noticed (Arning and Ziefle, 2009; Wilkowska and Ziefle, 2009). Furthermore, Wilkowska et al. (2021) conclude that health status and gender significantly influence opinions regarding technology acceptance associated with aging, emphasizing the correspondence of three factors: health status, gender, and age.

Lines (2000) states that the younger generation is more vulnerable and gullible to media messages and content, and its inability to consume sports media in a perspicacious way is concerning, along with their high sensitivity to sports celebrities’ influence. The age of a user is a crucial factor in the intensity of their mobile application use (i.e., the official application of a mega sports event) (Schut and Glebova, 2022); young event visitors (especially those under 25 years old) can easily install and use an application and benefit from its use. They post regularly and quickly navigate mobile applications to find necessary information. By contrast, visitors older than 50 do not actively use mobile applications and find them difficult and useless. They rarely follow or share information on the web. Vogels (2019) clarifies the differences between generations in terms of smartphone use and employment, finding that young people much more easily adopt and use smartphones and related technologies when compared to those in the baby boomer and silent generations. However, Vogels (2019) notices that adoption rates for this group are growing rapidly. Also, older Internet users are less likely to view the Internet as a positive for society.

The findings of other studies suggest that technology acceptance is not correlated with age (Jaafar et al., 2007) and the relationships among perceived ease of use, perceived usefulness, and intention to participate in online communities do not change with age (Chung et al., 2010).

## Methodology

### Data Collection

It refers to the customer-centric approach in the spirit of grounded theory. It is based on the literature review and written qualitative, semi-structured interviews (*N* = 12) with sports marketing, management, and technology professionals. We used purposive sampling to target experts who work in the rapidly changing context and have a holistic view of the field. All written interviews (or immediately transcribed interviews) were followed by further interrogations, clarifying questions, and precisions. Notably, to improve the accuracy and viability of the interpretation the interpretive validity member check has been done during the data collection process. We have been using paraphrasing and summarization for clarifications to be sure that research participants’ viewpoints and thoughts are understood (and interpreted) accurately. Interviewees have spent 45 min on average for answering all our questions. All interviewees gave their written permission to use the information they provided. Interviewees also have been given sight of the summary of this article before giving approval.

The qualitative approach allowed us to gather in-depth insights into the problem and generate new concepts through data synthesis and analysis. Also, considering the transformational nature of the study topic, this approach and involvement of experts offered an opportunity to capture changing attitudes within the sports industry. Moreover, taking a qualitative approach to the research was useful since it is not bound by the limitations of quantitative methods and focuses on the primary questions of “how” and “why” the mass deployment and diffusion of digital technology has transformed the consumption of content in the sports industry and affected audiences from different perspectives.

The research questions asked the following:

• What is the role of massively disseminated digital technologies in sports spectacle culture and notably in SSCX?
• How is digital technology interrelated with the process of sports-spectating audiences’ transformation? What are the key dimensions of technological influence?
• Why can digital technology mass deployment be considered the main driver of sports-spectating audiences’ change on a global scale?

### Data Analysis

We reviewed the literature and identified the preliminary key constructs to shape the theoretical background of the current article and create the basis for developing the interview questions. The data analysis included several stages. First, we systematized and inductively coded the collected data, employing a ground-up approach and deriving main codes from the data. We attempted to categorize all preliminary codes and figure out how they fit into a provisional coding frame. To this end, responses fell into two main categories: flexibility (consumer ability to access sport content regardless of time and location, mobility) and inclusivity (diversity, making sport spectating audiences people with various identities, demographical characteristics, and statuses).

Second, we iteratively read all the materials (fragmentally and entirely) several times and outlined the key points, employing thematic inductive manual coding. We went through the data line-by-line to elaborate on all textual materials as much as possible and get more detailed codes. At this stage, we identified merging codes and paid attention to interrelations between key categories and subcategories in an attempt to establish a kind of taxonomy (to be used as a provisional system for further building a new theoretical framework in the current study). Later, based on the iterative analysis, we synthesized all the theoretical and empirical insights and findings in order to achieve the results. Finally, the hierarchical coding frame organized codes based on how they relate to one another.

We have been continuously engaged in the process of reflexivity throughout the research process, constantly evolving in our understanding of the problem. Recently, we have conducted a series of similar studies, thus, tried to be free of *a priori* assumptions and definitions.

## Results and Discussion

The results and discussion are combined because the results are more valuable if directly followed by interpretation from the outset. All the interviewees firmly acknowledged and avowed that the global face of sports-spectating (consuming) audiences is transforming. However, they have observed various factors, perspectives, and dimensions explaining this process of change. Alex Montreuil (interview) explains this transformation according to the rise of three factors: (1) the impact of COVID-19 on the organization of events in the world of sport; (2) the influence of digital technology on consumption patterns; and (3) the perspective and reflective aspect of the future of the event industry. Moreover, Florian Lefebvre (interview) argues that “the growth of e-sports spectating completely transforms traditional sports global audiences. But more than e-sports, it’s the global changes in terms of how the user consumes information online that foster these changes with social media and streaming platforms that tend to replace traditional media such as TV or radio.”

Sport is changing quickly (Poll, 2019), and the pace of this transformation is being accelerated by the dissemination of mass technologies, the deployment of innovations (Glebova and Desbordes, 2021), and the COVID-19 pandemic (Glebova et al., 2022). Overall, automatization and digitalization both have an evident positive impact on sports spectating (Poll, 2019; Glebova et al., 2022); however, it seems impossible to evaluate nowadays (Wong et al., 2020). Measuring the change and transformation of audiences is still a problem for the industry (Cristian Gheorghe, interview) and seems to be a perspective research direction for the foreseeable future. Nowadays, technology is “all-pervasive” and sport is “something perfect for people to film and share” (Kevin Rye, interview). The mass deployment and diffusion of digital technology have transformed content consumption in the sports industry as it has created new channels and new ways to connect with fans beyond the sporting event itself. More importantly, it brings fans closer to the game so they are able to watch from different angles and analyze the game more closely (Amr Elrawi, interview).

Using digital technologies (e.g., mobile apps and social media), audiences can now follow sports performances in real time through streaming, thereby making sports consumption financially affordable and accessible from anywhere:

Mobile, data, and technology have transformed how fans are consuming sports and how the game is played. Sitting in a room watching sports is not appealing anymore to many people, especially the younger generation. Fans want to be closer to the game, augmented reality (AR) and virtual reality (VR), data, and game stats. We also see technology is part of the game now to identify in real-time by fine margins the winners and losers of many games (Amr Elrawi, interview).

According to Ciprian Enache (interview), sports spectators are not traditional consumers because sport means passion and sometimes defines the spectator’s sense of identity. Emerging technologies are changing how teams, leagues, and other entities around them create new fan experiences inside or outside of stadiums (Ciprian Enache, interview). Social networking sites first emerged as online public spaces where individuals, including sports-spectating audiences, share user-generated content, communicate, and connect (Campbell, 2018).

The synthesis of theoretical and empirical inputs has shown that there are two main factors behind changes in global sports audiences as impacted by the rapid uptake of digital technologies on SSCX: flexibility and inclusivity.

### Flexibility

Digital technologies offer a certain level of flexibility when it comes to watching and following sports through two main dimensions: place and time. It can be seen as consumer ability to access sport content regardless of various circumstances, benefiting from mobility and connectivity.

The Internet and mobile technology have meant that content can be broken up into any number of different formats and offerings, and social media makes it possible to share this content around the world (Kevin Rye, interview). This fact allows personalized content and services to be offered, thereby enhancing SSCX.

Furthermore, the Internet and mobile have given rights holders access and channels to connect with all fans from around the world, which creates amazing opportunities to monetize global fans. The sports business model has been transformed by technology and data. By shifting the focus from local fans to global fans, from linear broadcasting to over-the-top television, and from retail stores to e-commerce—and not least, e-sports—sports brands have found new ways of connecting with fans (Amr Elrawi, interview).

#### Place

Using mass dissemination technologies, spectators are able to access sports products regardless of their geographic proximity (place, address). Through technology, fans can follow sports teams from other countries (Pu and James, 2017), and online social communities (Wagner et al., 2017; Wagner, 2021) allow fans to be part of a group without having a physical presence in a particular geographical location. Place (a physical location of content consumer) does not matter; however, engagement and connectivity matter instead. It is a reason of raise of sport spectating global audiences, including distant, satellite, and displaced fans.

By taking a holistic approach and considering the trend of the globalization of sports fandom, this study focuses on the global audience, seeing it as an entire target sports-consuming population (Harrington and Bielby, 2007; Harrington et al., 2011). However, we cannot neglect the fact that the interviewees widely opposed the concept of geographical zones (e.g., setting Europe against the United States in terms of SSCX digitalization and audiences’ digital transformation as well).

#### Time

Digital technology boosts the speed of real-time information being shared between fans (Cristian Gheorghe, interview) through social media communications, individual and collective communications, and personalized and non-personalized communications as well (Samuel Lopez, interview). It means that nowadays the variety of sport content is accessible for global audiences at any time. The 24/7 accessibility of all types of sports content in different forms and formats makes sports consumption easier and more flexible, promoting the diversity and inclusivity of fans (Glebova et al., 2020; Stijn Jacobs, interview).

### Inclusion

The reason for extensive media consumption and the influence of modern digital communication is that the traditional concept of identity is fading (Cristian Gheorghe, interview; Samuel Lopez, interview; Axel Montreuil, interview). Consequently, newly important cultural norms and dynamics should be taken into account in terms of the tools, language, and imagery used in customer communications (Itonics, 2021; Ciprian Enache, interview; Samuel Lopez, interview). This is coupled with an “audience problem,” as social media users are unable to determine who is receiving information that is shared through social media (Hampton, 2016). This can create difficulties in determining the size and nature of audiences and attracting and focusing the attention of specific actors (Hampton and Wenhong, 2021; Kevin Rye, interview):

Digital technologies make it easier to give visibility to groups or initiatives that previously did not have access to distribution channels. Now that has changed, with social media being a very effective channel. The use of hashtags is a clear example. In the end, what technology itself allows is to offer more diverse services, but the final impact will depend on how managers decide to use, or not, these possibilities (Samuel Lopez, interview).

Digital technologies make sports watching more inclusive, providing “more angles to the diversity” (Cristian Gheorghe, interview). At the same time, the “multidimensional me” concept (Itonics, 2021) blurs consumer targeting and segmentation. A current trend in modern and connected society shows that sports fans’ individual self-identification and determination is changing, thus leading to the appearance of new groups of people and ideologies (Stijn Jacobs, interview). Consequently, a more complex, diverse, and multicultural sense of identity is emerging that embraces a wide range of dimensions, among them are nationality, race, sex, sexual orientation, religion, and myriad other facets of human identity.

#### Age

Meier et al. (2015) propose interpreting consistent evidence on age effects as an indication of the positive impact of less restrictive gender socialization on female fandom. Montreuil (interview) observes a “generational gap” in sports consumption and the impact of digital technologies on sporting events audiences, stating the following: “Technological disruption is giving new perspectives to start-ups to develop new concepts for new generations.”

The mass deployment and diffusion of digital technology has transformed content consumption in the sports industry simply because the generations Z, X, Y lacked the inputs to match their consumption patterns. The old generation needed visual entertainment, which correlated with the fact that digital technology was not yet at its peak; today, the trend is the opposite. The rise of new technologies allows for a stronger interest in sports consumption, and a plethora of technologies evolve every year: “I am part of the Z generation, which is highly digitalized; however, the alpha generation, which is coming, will be even more so. There is talk of gamification in the future with processes such as the hologram, the blockchain, or the possibility of using artificial intelligence (AI)” (Axel Montreuil, interview).

As the world of sports faces an aging fanbase, gamification will play and is playing a big role in attracting younger generations. One of the objectives of gamification is to reach as many fans as possible—locally and globally—and bring them closer to the game. This can only be achieved by adopting digital transformation (Amr Elrawi, interview):

This shows the interest of new audiences in practicing gamification in sports, it is the interest “phygital,” the new generations wanting to practice physically no longer want to practice in a club or an association judged to be “rigid” by its mode of operation. This would make it possible to restructure the practice of sport and above all to adapt it to its audiences. In France, we have a tendency to let young practitioners slip through the cracks, yet we know that they represent nearly 60% of the members of federations (aged 6–24). This is why some federations have understood the interest in associating themselves with the gamification of practices through start-ups (Axel Montreuil, interview).

The growth of e-sports affects traditional sports audiences and can be considered a competitor of traditional sports consumption (Florian Lefebvre, interview). However, this argument has another side, as Amr Elrawi (interview) explains:

If you have an hour to spare, you have a choice today to go play sports, watch a live game, or play an e-sports game, or even order pizza and watch a movie. In the experience economy, brands are not necessarily competing with direct/traditional competitors. They are competing against the share time and wallet of customers.

In the framework of the constant and complex transformation of sports audiences, older generations can be left behind due to lower levers of technology acceptance, perceived usefulness, and perceived ease of use because cutting-edge technological products and services do not often appeal to older generations, targeting “zoomers” instead. For example, generations X and Y do not want to discover more technology for fear of “infobesity” and are unfamiliar with many emerging innovations (Axel Montreuil, interview; Ryan McCumber, interview).

#### Gender

Even though technology is allegedly gender neutral, women and men have been found to fundamentally differ in terms of their communication patterns (Koinig et al., 2020). Sports spectating experiences cannot be an exception in this regard (Andrei Angelescu, interview; Ciprian Enache, interview).

Meier et al. (2015) refer to “feminization” in the sports fandom context as it applies to the increasing commercialization of sports and changing gender roles. Moreover, the “multidimensional me” trend affects gender and sexuality dimensions, but sports fans can be considered a specific group of consumers (Ciprian Enache, interview). Analyzing the relationship between gender, sexuality, and fan identity, Allison and Knoester (2021) find that women and non-binary adults are less likely to identify as strong sports fans when compared to men. Compared with identifying as heterosexual, identifying as lesbian, gay, bisexual, or another sexual identity is negatively associated with self-identified sports fandom. Also, gender and sexuality interact such that identifying as gay (or lesbian) is negatively associated with men’s self-identified sports fandom but not women’s fandom (Allison and Knoester, 2021).

#### Health Status/Disabilities

The fact that mediatory watching gives people with limited physical and mental abilities an opportunity to consume sports is far from a new discovery, and it often turns them into sports followers and fans. Furthermore, with the rapid development of various technologies in sport, this opportunity is becoming enhanced (Andrei Angelescu, interview; Amr Elrawi, interview; Ryan McCumber, interview; Ciprian Enache, interview). It goes beyond mediatory watching and extended access to information, but it is easier for people with special health status or disabilities to operate and navigate in-stadium as well in terms of infrastructure and connected services.

As a sports leader working with people with intellectual disabilities, I know that the pandemic situation in recent years has resulted in the exclusion of many people with intellectual disabilities. Mostly because they weren’t allowed to play sport and socialize. Special Olympics has therefore launched a number of online sports programs which aim to enable athletes to exercise online. Using exercises at home and social media, people reached out to each other and stayed in touch but there were still severe losses due to the lockdown. In my opinion, the global sports consumer has also been exposed to many new opportunities that have allowed them to maintain their interest in sport (Kata Orbán-Sebestyén, interview).

### Seeing Change as an Opportunity

Sports-consuming audiences are gradually being reshaped in various ways. The number of devices and technological tools used in SSCX is continuously growing. On the one hand, this may bring uncertainty, but on the other hand, this may offer new marketing and management opportunities that could be turned into an advantage by sports managers and other stakeholders. First of all, technological products and services must be user-friendly, easy to exploit, and associated with being useful in terms of perceived usefulness and perceived ease of use (Davis, 1989). According to Cristian Gheorghe (interview), “the usefulness and perceived ease of use are translating in time of transformation. Hard means slow transformation.”

Perceived usefulness and perceived ease of use affect this process of transformation. It has a big impact; sports brands need to focus on business outcomes, not on technology. Also, it is important to take the whole organization on a journey with a clear long-term vision and short-term wins so people can see progress. Digital transformation is a process to improve [the] customer experience and drive commercial value; this can only be done if the organization [is] focused on four pillars: people, process, data, and technology. Sports brands that don’t embrace technology and data will be out of business within 10 years. Data and tech will deliver and maximize the commercial value of all sports brands’ products (Amr Elrawi, interview).

Gamification and e-sports trends should be followed and considered by traditional sports structures (Florian Lefebvre, interview; Ciprian Enache, interview; Amr Elrawi, interview), becoming a part of SSCX strategy. The growth of e-sports spectating can be a source of inspiration for traditional sports spectating in the sense that the latter trend is now more inclined to communicate/broadcast new types of content on streaming platforms like Twitch, for instance. Some clubs have already aligned their traditional football communications with social media on e-sports codes by hiring people with professional e-sports backgrounds to carry out this task for the whole football club (Schmidt and Holzmayer, 2018; Florian Lefebvre, interview).

The “multidimensional me” trend and easier access to sports content are influencing the range of products and services that modern spectators find relevant and valuable, thereby shifting both social and cultural perspectives of SSCX and blurring the traditional understanding of targeting and segmentation concepts. Subsequently, sports marketing professionals face the complex task of adjusting products and services to appeal to diverse identities, focusing on inclusion, integration, and personalization rather than adoption, assimilation, and “digestion” (Itonics, 2021; Ciprian Enache, interview).

Furthermore, Samuel Lopez points out the importance of technological adoption from the managerial perspective by mentioning modern phenomena like addictions, fake news, and trolls, all of which are increasingly present in the digital environment. He believes that sports spectators should be trained to be prepared to process a large amount of information: “They must be able to analyze it critically before being influenced by the masses” (Samuel Lopez, interview).

## Conclusion

The face of the global sports spectacle-consuming audience is constantly changing, and the development and mass deployment of digital technologies is the main driver of change. This process has been and will continue to be accelerated by the COVID-19 pandemic. Accordingly, it requires further research and attention from scholars and sports management professionals. By synthesizing the literature and collecting data, we were able to attribute the transformation of audiences in terms of their age, gender, health status, levels of mobility and flexibility, diversity and inclusivity, and the fading of individuality to digital technologies. Moreover, as a tool and means of accessing sports content, technology enables fans to be connected at all levels (i.e., peer to peer, community, sports organizations, clubs, and with sports celebrities) and for SSCX locations to change (Glebova et al., 2020).

The implications of this research are complex and variable, leading a reader into the realm of speculation. From the theoretical point of view, it can be seen as an addition to existing theories (for example, technological determinism) and material for new theories regarding the impact of digital applications on sport spectacle and various facets of technological transformation through a customer-centric perspective. Thus, it provides relevant up-to-date profound insights and promising areas to work on. From a practice perspective, findings are useful for managers and policy-makers to better understand sport spectating audiences, trends, and processes in an ever-changing global society. It allows to deliver better customer experiences, and build effective and efficient customer relationship management strategies.

As for the limitations, this study is not statistically representative and cannot be repeated. Moreover, we did not disclose the number and scale of audience transformations. Although we measured these changes holistically from a qualitative perspective by outlining the main dimensions to acquire a helicopter view of the question, doing so prevented us from going into detail and gaining deeper insights. For future research directions, we suggest focusing on the proposed dimensions and investigating the nature and impact of every single facet of transformation. Finally, this problem should be investigated from a quantitative perspective in order to ensure it is statistically analyzed and represented.

## Data Availability Statement

The raw data supporting the conclusions of this article will be made available by the authors, upon request.

## Ethics Statement

Ethical review and approval was not required for the study on human participants in accordance with the local legislation and institutional requirements. The patients/participants provided their written informed consent to participate in this study.

## Author Contributions

EG, MD, and GG contributed to the conception and design of the study and wrote sections of the manuscript. EG organized the database, performed the analysis, and wrote the first draft of the manuscript. All authors contributed to manuscript revision, read, and approved the submitted version.

## Conflict of Interest

The authors declare that the research was conducted in the absence of any commercial or financial relationships that could be construed as a potential conflict of interest.

## Publisher’s Note

All claims expressed in this article are solely those of the authors and do not necessarily represent those of their affiliated organizations, or those of the publisher, the editors and the reviewers. Any product that may be evaluated in this article, or claim that may be made by its manufacturer, is not guaranteed or endorsed by the publisher.
